# Perceptions of Parenting during the COVID-19 Quarantine Period, in Suceava, the Epicenter of the COVID-19 Outbreak in Romania

**DOI:** 10.3390/ijerph192316188

**Published:** 2022-12-03

**Authors:** Marius Marici, Otilia Clipa, Remus Runcan, Iasmina Iosim

**Affiliations:** 1 Faculty of Educational Sciences, Stefan cel Mare University, 720229 Suceava, Romania; 2 Department of Pedagogy, Psychology and Social Work, Faculty of Educational Sciences, Psychology and Social Work, Aurel Vlaicu University of Arad, 310032 Arad, Romania; 3 Economics and Finance Company Department, Faculty of Management and Rural Tourism, University of Life Sciences “King Mihai I” from Timisoara, 300645 Timișoara, Romania

**Keywords:** quarantine, parents’ perceptions, COVID-19, children’s complaints, family activities

## Abstract

Recent findings suggest that quarantine adversely affects an individual’s wellbeing. Social isolation generally leads to many negative psychological outcomes in child development and to significant shifts in parent–child relationships. The aim of the present research was to investigate three aspects of parenting during the COVID-19 quarantine: what activities parents performed better during the quarantine, what difficulties parents had during the quarantine, and what complaints children had living in Suceava, a city that saw the greatest number of infected individuals as well as the largest death toll amongst all regions in Romania. The respondents were 201 parents from Suceava, Romania (M_age_ = 36.71, SD = 7.22), who answered a self-reported questionnaire after a quarantine period of between 30 and 33 days (30.04.20–2.05.20) concerning three qualitative questions and items related to parenting activity, parent–child relationships, or child behaviors. Among the key findings, the research found that parents had a good perception of their parenting skills during the quarantine time, the most difficulties reported by parents were about the newly imposed social realities, and the most obvious unmet need of children was the lack of social interactions. The findings bring to light the perception of forced time spent together by parents and children. Further research should investigate how parenting fluctuates in crisis situations such as the one highlighted in this article.

## 1. Introduction

The COVID-19 outbreak quarantine created a new and special parenting environment characterized by living together for tens of days and challenging parents to adapt to influencing children efficiently. Literature generally describes parenting in the narrow sense, through psychological dimensions such as behavioral and psychological control, parental support, autonomy granting, and clear communication or parenting styles [1]. Yet in the broad sense, parenting refers to more than that. The American Psychological Association describes parenting as referring to its aims such as: ‘ensuring children’s health and safety, preparing children for life as productive adults, and transmitting cultural values’ [2] (n.p.). The present study viewed parenting as referring to all interactions or actions between children and parents, initiated by parents or guided by them and for which parents felt responsible and were aimed at ensuring child socialization and multidimensional growth.

There are no studies to investigate parents’ perception of their parenting during the quarantine time in Suceava, the first quarantined city in Romania. Every city has its social, cultural, political, educational, or economical specificity, in which people are used to live. Quarantine as a crisis moment, involves mainly a reorganization of family life and might put additional pressure on children and parents to behave and feel differently. The outbreak details, the quarantine time, the isolation and lockdown effects, the shifts in parents’ roles, the adaptation to the new social imposed realities or the permanent parent–child time spent together during the quarantine bring changes in parents’ interactions and perceptions. Thus, our study intends to adopt a post-positivist method in order to find thematic categories regarding parents’ perceptions of their parenting, considering both positive and negative aspects, in the context in which most studies published during the quarantine time focused on the detrimental effects of quarantine.

## 2. Overview of COVID-19 Outbreak in Suceava

Suceava was, until the date of the present research, the only quarantined city in Romania and was for the country what Wuhan was for China, or Lombardy for Italy. The rapid unfolding of worry regarding the COVID-19 virus spread gave rise to the instauration of an emergency state on the whole territory of Romania on the 16 March 2020 (Presidential decree no. 195/16.03.2020) [3]. Soon after, the North-Eastern city of Suceava and eight surrounding areas (Adâncata, Salcea, Ipoteşti, Bosanci, Moara, Șcheia, Pătrăuţi and Mitocu Dragomirnei) were placed under quarantine, through a military ordinance issued by the Interior Ministry, beginning on the 31st of March 2020. Suceava has a population of about 160,000. Romania is divided into 41 counties (Ro ‘județe’). At the time of the research development, Suceava (one of the counties) recorded more than ¼ of the total confirmed infections in the country and the largest number of deaths, more than ⅓ of the national number [4].

Normal life activities were drastically limited and public circulation was stopped. To be as explicit as possible, the decree established that the lockdown does not allow citizens to move or transport from one place to another, and they must remain within the limits of their property. Yet, there were exceptions, and people had to complete an affidavit and include personal data, their address, the reason for mobility, date of filling in and a signature. In most cases, family members still circulated only if they had a firm or had to buy food or medical supplies. Citizens had to circulate within certain time intervals and had to take measures regarding personal protection against the COVID-19 virus (respiratory masks, medical gloves, using disinfectant gels or ethanol for hands, or maintaining a distance of 2 m from other people).

Suceava and the surrounding areas are well known for their religious heritage and painted monasteries, part of the UNESCO World Heritage, for rural tourism, and for preserving Romanian traditions.

## 3. Quarantine Time

“Quarantine is the separation and restriction of movement of people who have potentially been exposed to a contagious disease to ascertain if they become unwell, so reducing the risk of them infecting others” ([5], n.p.). In this context, family life continued at home 24 h a day. In the case of adults, they had the chance to travel from time to time, inside the area for emergency reasons, but children stayed permanently at home. The data for the present research were collected starting with the 30th day of quarantine and ending three days later.

Literature about quarantine time writes about several realities that are specific to this particular reality such as: forced proximity, physical or psychological isolation, lockdown, shifted roles or changes in the time spent together. On one hand, overall, quarantine periods are generally not considered pleasant events that produce desirable consequences but rather times of various interpersonal and social difficulties, for parents and children as well. Quarantine time often happens without previous warning and a priori preparation, and in certain conditions, it might provoke mild to severe psychological and social effects [6]. Research shows that quarantine resulted in separation from loved ones, who might be trapped in other locations, isolation, shut down of personal economic affairs, and rescheduling of family plans and personal life [7]. Studies indicated that physical isolation, which occurs in quarantine, is associated with internal problems such as anger, fear [8], anxiety [9,10], confusion, boredom, insomnia, dependency tendencies, physical performance [10,11], inadequate supplies or information, suicide tendencies, etc. [8,12]. Bai et al. [13] found that nine days of quarantine might lead to more serious psychological problems such as signs of acute stress disorder. Sprang and Silman [14] found that pandemic disasters create traumatic conditions for family members. Forced proximity is a risk factor for aggression and domestic violence [15]. In a very recent review of 24 studies [7], the results indicated that quarantined people generally report detrimental psychological effects. Stay-at-home time in a crisis such as the SARS-CoV-2 might be associated with negative psychological effects but also with social and economic negative consequences [6].

Indeed, few studies describe that family members performed better during quarantine than before. Few studies find clear evidence that the pandemic produced positive psychological effects. People like to cooperate when they know that other people are cooperating [16], and they prefer to focus on local charity, for example, rather than on global charity, although because of social distancing, empathic concerns and lack of perceived opportunities dropped considerably [17]. In a context of crisis, it is paramount to grant more time to self-care, help the needy surrounding us, and support family needs. All these essential actions strengthen the self, the family, and the community [9].

Isolation made people adapt and find creative solutions to keep communication going and to develop social interactions at novel levels, through virtual platforms such as Zoom, Google Meet, Cisco, Microsoft Teams, etc. In Romania, the pandemic found parents and their children unprepared for online interactions and forced them to acquire intensive skills and abilities to save the school year [18,19]. The pandemic likely made families have closer relationships, and made parents, who could no longer could claim to not have time, make better use of their functional parenting skills [20].

## 4. Isolation and Lockdown

Children isolated indoors have fewer ways to maintain a satisfactory social life. Interactions with their siblings and parents remained the only option available. Social isolation is generally related to negative psychological outcomes regarding the development of children and to shifts in parent–child relationships. Physical distancing, which refers to the lack of social interactions, is associated (in the research literature) with a growth in the number of psychological and medical pathologies due to job insecurity or poor expectations about the course of personal life and the development of society as a whole [8,10,21].

In a recent review, the results indicated that the quarantine period had a negative effect on wellbeing [7]. Another study revealed that adults in Romania felt boredom and in a “prolonged context of COVID-19 social isolation, boredom completely mediated the relationship between coronavirus anxiety and aggressiveness” [22] (p. 85). Family communication was expected to increase during the lockdown. Sherman [10] showed that close family communication correlated with a low score on the Coronavirus Anxiety Scale [23]. Sitting together for a longer time, in the same place, might be an additional stressor to family life that could escalate into conflicts [24]. Isolation restricts meeting with other people, and the lack of social contact will reduce the chance of the transmission of respiratory infections such as SARS-CoV-2.

## 5. Shifted Roles and Adaptation to the New Realities

The pandemic led parents to modify their roles during the lockdown compared with the time before. Children spent most of their time adapting to a new environment that had been unknown to them until then. These new challenges triggered significant educational transformations, one of them being the shift from onsite to online learning [18,25]. The roles of teachers, parents and children were changing in that special context. Children in Suceava lived mostly indoors doing online school lessons on platforms such as Adservio or connecting with peers and teachers through online streaming applications, in a context of skepticism regarding the efficacy and success of online learning. Parents took on the role of home-schooling overnight, without previous training, and were forced into becoming supervisors and teachers for their children. As if that was not enough, the Ministry of Education in Romania issued ministry order no. 4135/21.04.2020 [26], which states that it is mandatory for pupils to attend online courses, not taking into account that there was a problem with the lack of access to the internet and computers for students. Furthermore, few teachers attended in-service courses for implementing modern technology in education [27]. This added much more pressure on parents and students, especially those living in rural places and those who had more children because they were younger or had a lower socioeconomic status [28].

## 6. Parents and Children Spending Time Together during the Pandemic

Time spent together might be a drawback or an advantage. Time spent by parents together with their children is a classic already in parenting literature. Today’s parents spend much more time with children than decades ago [29]. Two possible reasons are that fathers increased their time with children and families have fewer children. However, fathers spend much less time with children than mothers [30]. From this viewpoint, the pandemic might have been a unique opportunity for them to know their children better and be, even if for a shorter time span, full-time fathers. In addition, fluctuations in parental conflicts, due to time spent in isolation with family, might amplify or decrease negative effects associated with marital conflicts [31,32]. These variations might lead to variations in parental perception of the affective environment of family life or could influence parent–child relationships.

There is little evidence in research that intensive parenting, with large amounts of time spent by mothers with children, leads to better child outcomes [33]. A study found that the fact that mothers were accessible to children or that they engaged with children was not associated with significant externalizing or internalizing problems and did not lead to higher scores in math or reading. [34]. On the contrary, further evidence suggests that spending time with children can be stressful and tiresome, and in some circumstances, positive effects might be scarce [35]. From another viewpoint, spending time together is widely recommended for all parents. Quantity time might be for quality time, what brain is for the mind, a basis and support. Even if much time per se spent with children does not produce a direct effect on child outcomes, in various areas of child development, spending time with children might be a strong moderator than the cause or a mediator between parents’ actions and child outcomes. In addition, quality time “includes all activities in which either the child was the primary focus of the activity or in which there would be a reasonable amount of interaction” [36]. Thus, quality time is better defined when children perceive their interaction with their parents as positive and pleasant. In this way, quality time could be an indicator of parental love, a basis for a variety of parenting features to develop, and/or a forced necessity as it happened in the COVID-19 quarantine, although some overlapping between these could appear.

## 7. Research Methodology

### 7.1. The Present Research

Suceava and its surroundings became the epicenter of the COVID-19 outbreak in Romania, with the most infections and deaths. The decision to totally quarantine the city of Suceava and its surroundings, in an attempt to stop the infections, represented a natural experiment that provided us with unique data for scientific inquiry. The study investigates the perception of parenting from inside the SARS-CoV-2 outbreak and the effect of total isolation of families at home for at least 30 days.

The aim of the research was to qualitatively investigate parents’ perceptions of some parenting aspects during the pandemic regarding three topics: (1) what activities parents thought they performed qualitatively better during the quarantine that they could not do well or at all before, (2) what difficulties they had during the quarantine period as a parent, which they did not have before the quarantine period, (3) what were the most frequent complaints verbalized by children during the quarantine period. The research adopted a post positivist method [37], combining qualitative results with descriptive quantitative analyses and investigated parents’ perception of their parenting in a context in which they changed family roles during the quarantine. Moreover, the present research was more interested in finding activities performed better, difficulties faced, or complaints verbalized by children rather than understanding and depicting the whole semantics of parenting life during the quarantine.

### 7.2. Measures

Parents were informed in writing that the study was about understanding their subjective perspective on activities realized during the quarantine time, difficulties felt, and complaints of children, as viewed by them from their role as parents. The parenting concept was defined for parents as referring to all interactions or actions between children and parents initiated by parents and guided by them and for which parents felt responsible and aimed at ensuring child socialization and multidimensional growth during the quarantine. Our study did not investigate the entire family life during the lockdown, although there might be a major overlap; it was interested only in the parent–child subsystem. As the present research is a qualitative study, respondents were free to refer to whatever they interpreted that parenting might include.

The qualitative investigation asked respondents to indicate the following: (1) describe activities they performed qualitatively better during the quarantine period that they could not do well or at all before that related to parenting, (2) describe difficulties they had during the quarantine period as a parent that they did not have before the quarantine period, and (3) describe the most frequent complaints of children during the quarantine period. Respondents could openly answer these questions. Before collecting the data, 27 students took part in a pilot study and filled out questionnaires. They provided feedback regarding the content and form of the questionnaire. Their feedback helped us calibrate semantics and reach a final item form.

Participants also responded to demographic and personal questions to provide a more detailed characterization of the sample: place of residence, age, sex, type of dwelling, health status, financial status during the pandemic, existence of major stressors other than SARS-CoV-2 infection, education level, and the number of children living in the dwelling.

### 7.3. Participants

The participants of the present research were 201 parents, from different families (M_age_ = 36.71, SD_age_ = 7.22) who filled out online questionnaires via Google Forms, after they were shared on social media on pages or groups in the quarantined area of Suceava. Thus, most respondents were from Mitocu Dragomirnei, Ipotești, Bosanci, Patrăuți, and Suceava. The questionnaires took approximately 10 min to be filled out.

The data states that 86% of the respondents stayed with their partner, and 87% were mothers. By dwelling, 68% of them lived in houses with a yard and the rest in an apartment. Of all respondents, 73% had access to green spaces, and in each dwelling, there was an average of 4.2 people living together. Parents’ reports indicated that 88% of them were not sick within the quarantine period, and 75% did not report any major stress during the same period. Most parents (49%) had just the money they needed, 43% had a sufficient amount of money during the quarantine, and only 8% did not have enough money. Most parents (58%) had graduated from a faculty or had a master’s degree, and 42% had graduated high school or had a lower level of education These families had between one and seven minor children living in the same dwelling (M_no_children_ = 1.60, SD = 0.99). Parents filled out the questionnaire referring to school-age, minor children (6–18 years old) living with in the home and being present in the same dwelling during the quarantine period. The questionnaires were filled out between the 30th and the 33rd day of quarantine. There were five invalid questionnaires (from the total of 206), owing to lack of data, that were removed from the research. Thus, the valid number of participants was 201.

### 7.4. Data Collection and Analysis

The present research is a unique investigation of aspects of parenting in a quarantined city threatened by SARS-CoV-2 pandemic infections. The participants’ responses were collected voluntarily, online, after posting on social media, especially in Facebook Groups in the targeted quarantined areas. All authors distributed the questionnaire, and there was no identifiable relationship between the respondents and the researchers. All four researchers have previous experience in qualitative research. Each of the eight areas from Suceava has Facebook Groups, which are generally administered by a mayor or delegates who make announcements and send vital information to citizens. All answers regarding the present research were collected using a web-based, self-reported questionnaire.

The present paper adopted rigorous scientific procedures [38]. All qualitative data were coded by three independent coders who discussed the meaning units and their attribution to different categories and subcategories. The coders had at least a college degree in psychology or educational sciences. All the coding took place on paper or/and on personal computers. The mean inter-coder agreement was 0.92. In total, there were 1847 answers collected for the three research questions.

After collecting the questionnaire-based data, a manifest analysis was performed focusing on what was observed in the raw answers. The analysis adopted an inductive approach, bottom-up, where all codes were derived from our data. All codes were derived and permanently improved, regarding their formulation, during the coding process.

**Figure 1:**
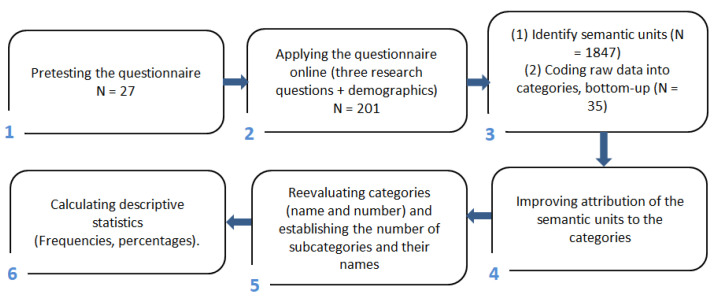
A flow chart indicating how the research was conducted.

Thus, the first step was to identify singular meaning units in the data, based on the inquiring questions. A “meaning unit” is the smallest piece of information that answers the questions set in the questionnaire [39]. Often, more relevant information was coded into the same clause, which made assessors attribute them to different categories. The second step was to read again all meaning units carefully and, based on the mental processes of distancing and approaching (re-contextualization), ensure that all semantic units were grouped correctly. The third step was to set up categories. They are semantic units that include all related meaning units. Then, subcategories were created that included meaning units grouped within each category. Moving back and forth, a singular meaning unit between categories and comparing and contrasting them ensured their belonging [40]. The fourth step was to calculate descriptive statistics, percentages, and frequencies when necessary (see Figure 1).

### 7.5. Ethics of Research

All participants filled out the forms after finding the post on Facebook and after reading an informed consent. They were informed about the people in charge of the research responsibilities, confidentiality, purpose, how their data would be used, the institution leading the research, and details about the people in charge of the research. Informed consent was obtained from all participants in the research.

## 8. Results

The aim of the present research was to better understand some aspects of parenting by finding out from participants what activities they performed better during this period, what difficutlies they faced during the quarantine, and what complaints children verbalized, not to understand the whole semantics of parenting life during the quarantine. Thus, participants’ answers varied from short and on the topic to longer answers that included some explanations. As the research interest was to find activities, difficulties, and complaints, a large number of answers (*n* = 1847) was generated that could be classified into categories and subcategories. Although the data were qualitative because they were specific, it was easier to calculate some quantitative results such as frequencies and percentages for a better understanding of the answers.

### 8.1. Findings for the First Research Question—What Parents Think They Did Qualitatively Better during the Quarantine Than before?

Firstly, parents were asked to describe activities they performed qualitatively better during the quarantine period that they could not do well or at all before, referring to parenting responsibilities and duties. There were 546 answers grouped into multiple categories (Table 1):

### 8.2. Findings for the Second Research Question—What Difficulties Did Parents Have during the Quarantine That They Did Not Have before?

Secondly, respondents were asked to name difficulties they had during the quarantine period as a parent that they had not had before the quarantine period. There were 525 answers grouped into 16 distinct categories (Table 2).

### 8.3. Findings for the Third Research Question—What Complaints Did Children Verbalize the Most Frequently, during the Quarantine?

Finally, parents were asked to describe the most frequent complaints verbalized by children during the quarantine period. The results indicated ten categories and 776 individual answers (Table 3).

## 9. Discussions

The aim of the present research was to investigate aspects of parenting in the context of a natural experiment, the quarantine time in Suceava caused by the COVID-19 pandemic. This is definitely an exceptional circumstance and not an ordinary everyday normality.

### 9.1. Discussion of the First Research Question—What Parents Think They Did Qualitatively Better during the Quarantine Than before?

Of the parents participating, 97.07% reported a qualitative increase in the activities mentioned above, during the quarantine time: as a vast majority of parents indicated that personal schedules and roles changed drastically. Activities dealt with are always connected to the respondents’ lifestyle [41,42]. As there was a major cessation of all major regular, daily activities and family members lived at home, most activities that parents performed better than before were free time activities. In addition, domestic chores occupied about 20% of the total activities. Families adapted and adopted novel activities such as exclusive online schooling or learned new skills regarding hygiene against infestation with COVID-19. These last two categories indicate shifts in parental roles owing to the quarantine situation and were presumably directly triggered by education campaigns and public health policies. Categories such as money management or hygiene activities regarding COVID-19’ seem not to be related to parenting at all and time for self or reading subcategories seem to be personal. Yet before drawing such a firm conclusion, it is important to remember that the research investigated parents’ perception of parenting in a critical context, that of an overnight quarantined city. This probably elicited mixed emotions and a drive to preserve and conserve resources and survive in a context in which the risk of infection and death was a constant presence. We speculate that parents might have interpreted differently the issues during the quarantine as compared to the period before the pandemic. Going back to the raw responses, parents’ answers indicated that they initiated some actions (e.g., ‘I encouraged my boy to pray more.’ Alina, 34 years.) or even guided them (‘I made a schedule for the children to read more books for school.’ Marius, 41 years.), even if they are considered individual activities. So, even if children performed some activities alone, they were initiated or guided by parents, and parents attributed the merit for these actions to themselves and considered them to be some parenting issues. Relationship preoccupations and religious activities also increased in quality according to parents’ reports.

Reported data indicate that parents had many indirect opportunities for parent–child relationship improvements, especially during free time activities or chores, but also during religious activities or online schooling (77.82%), which all increased qualitatively during the quarantine period. Parents also mentioned that they consciously worked to improve aspects of the parent–child relationship (13.53%), as the context and time were sufficient.

Our analysis indicated that parents had a good overall perception of their parenting in the quarantine period, and parents perceived that time spent together had a positive effect on parent–child behavioral improvements and interactions. These findings are in accordance with other findings from other cultures. A recent study in Chinese and Turkish cultures found that two positive experiences during the quarantine period were “doing activities together” and “improvements in family relationships” [43] (p. 29). Probably some parenting aspects improved once given much more time than before, while other parenting skills remained undeveloped as they did not have much time to grow and be applied effectively, even when parents retained the skills. However, the present study investigated parents’ declared perceptions of parenting and did not investigate cause–effect inferences.

### 9.2. Discussion of the Second Research Question—What Difficulties Did Parents Have during the Quarantine That They Did Not Have before?

Parents reported the most diverse answers grouped into 16 categories. Most novel difficulties in parenting referred to adapting to the newly imposed social realities (37.85%). The new realities included social distancing or the interdiction to move outside the home and, even if at a lower rate, respecting norms referring to COVID-19. Within this category, it is assumed that most difficulties occurred because family adaptation involved directly or indirectly social distancing and the shutdown of all non-familial interactions. Humans are innately social. A meta-analysis (*n* = 308.849) found that having poor or insufficient social interactions puts individuals at about a 50% greater risk of death than those with adequate social relationships. The magnitude of this effect is comparable with quitting smoking and it exceeds many well-known risk factors for mortality (e.g., obesity, physical inactivity) [44]. A study investigating the negative experiences during the quarantine period found somewhat similar results, indicating that adaptation was difficult when referring to child discipline, screen time, social isolation, shifts in roles and routines, parent–child relationships, or managing child discipline and behaviors [43].

Parents also reported difficulties in emotional regulation in children (12.7%). Brooks et al. [7] found that there are multiple negative psychological outcomes as a result of being quarantined, such as depression, low mood, anger, emotional exhaustion, or emotional disturbance. In addition, parents found it difficult to motivate children to comply (7.23%) or to manage relationships with children (3.04%). Families were rarely together 24/7 for long uninterrupted periods before the quarantine, leaving parents in charge of managing challenging children by themselves. These findings are in line with recent studies that showed that parents found it hard to manage child tantrums, discipline strategies, or misbehavior of children during the quarantine period [43].

Nearly 10% of parents (9.71) reported online schooling difficulties, most of them referring to technical issues, lack of multimedia devices, or lack of training and experience. The sudden shift from onsite to online schooling in Romania, without prior training, might have been responsible for parents’ lack of skills and their difficulties.

### 9.3. Discussion of the Third Research Question—What Complaints Did Children Verbalize Most Frequently, during the Quarantine?

The answers to this question generated the most answers, 776, and the most verbalized complaints referred to the children’s need for socialization (46.93%). At a closer look, children who complained about the interdiction to move outside their property referred to places populated by many people, which suggests that this category has a strong social interaction function too. Indeed, a vast majority of children (69.45%) complained about the cessation of their social life. As the quarantine period included limiting movement and interactions with people, it was to be expected that children would have experienced a decrease in pleasurable social dynamics, which was expressed in complaints.

The transition to online schooling in Romania, without any pre-quarantine preparation, led to a series of problems faced by children. The panic to avoid the freezing of the school year led authorities, school principals, and teachers to require overnight shifts to online schooling without any inventory of the necessary material resources. There were many cases where there was no internet connection available, and children did not have a special place in the dwelling to organize ongoing school activities. Thus, family members felt pressure owing to the alert educational changes, and children found it difficult to adapt.

Although emotional problems are among the first described in literature when the family is in isolation, our research found, based on parental reports, that only 4.68% of the total verbal complaints referred to emotions. Parents’ results indicate that there was a greater diversity of complaints listed than things parents performed qualitatively better: parents tended to agree more on what challenges their children faced than on the activities they improved.

Some parents (2.19%) reported no complaints from the children, instead indicating that children managed the time in isolation well. This percentage could be so low because certain children had complaints but did not express them. However, recent research [7] does indicate that about 5% of quarantined people reported feelings of happiness and about 4% feelings of relief.

### 9.4. Discussing the Research Findings as a Whole—How Did Parents and Children Adapt during the Quarantine?

Taking a closer look at the data, if we find the common topics from each set of categories from each research question, the following can be inferred:

Firstly, although 38% of parental difficulties refer to having a hard time adapting to the new social pandemic reality and about 46% to 69% of the children’s complaints refer to restricted socialization, parents reported an increase of at least 47% in free time activities. Similarly, 1.33% of parents reported a decrease in religious activity. The most, 4.57%, reported an increase in religious activities at home, while only 1.02% of the total complaints were about the cessation of all church activities or even about too many religious activities. The balance may have been hard to maintain, as quarantine is a special time. It is very likely that in cases where it was possible, parents tried to compensate for the loss of privileges they had before the quarantine. Spirituality and pleasant activities are functional ways of coping with quarantine time as they might improve personal and family well-being [45].

Secondly, about 2% of parents stated that they faced difficulties with home chores, and about 20% of parents reported that they managed chores better during the pandemic. However, parents reported that children did not complain at all about chores during quarantine. The pandemic brought home chores for the first time with enough time for doing them.

Thirdly, more than 7% of parents could not effectively motivate children to comply with their directions, and about 3% did not succeed in managing relationships with children. Yet almost 14% reported that they granted special attention to improving relationships with children, while children did not complain at all about the issue of parent–child relationships. Parent–child relationships are a multidimensional reality that parents and children perceive differently based on their experience and social roles. However, the quarantine time was, for sure, a challenging period, especially if parents lived indoors with early adolescents [42,46].

Fourthly, approximately 10% of parents reported discomfort with online schooling, while children reported the most complaints (about 14%). Only 6% of parents reported successful adaptation to online schooling. Therefore, it seems that both parents and children were caught unprepared for online schooling. However, the fact that only 10% reported challenges related to online schooling may result from a somewhat biased sample, since people were only recruited from Facebook, which suggests all participants had internet access in some form.

## 10. Conclusions

The aim of the present research was to qualitatively investigate parents’ perception of their parenting during the quarantine period in Suceava, Romania. This is one of the few studies that depicts aspects of parenting in quarantined families after a period of 30 to 33 days of COVID-19 lockdown and also one of the few studies released by researchers inside the quarantined area, which shows how forced time together impacts family life in Romania. There are also few studies that found positive effects of the quarantine time, as did the present one. The quarantine time provided valuable data for investigating time spent together as a family.

The qualitative research results indicate that parents and children found constructive activities to pass during this challenging period and focused on spending more time in free time activities, chores, or social interactions than before the quarantine. Parents also found it more difficult during the quarantine time to maintain social dynamics, manage emotional regulation, deal with school issues, or motivate children to comply with parental norms. More importantly, children complained mainly about the lack of adaptation to the new social interaction norms.

As the quarantine period passed away, this study indicates that parenting is a complex and permanent process of adaptation and functioning at different levels, and this was better seen in the face of a novel situation such as the COVID-19 quarantine. Furthermore, the present study reveals how Romanian institutions can better meet such crisis situations and how they can better prepare parents through education courses or other available resources. The present study represents parents’ subjective voice from a quarantined city, with their perceptions about different parenting aspects.

One limit of the present research is that the data were collected based only on online self-reports and reported just by parents. It is highly likely that the results would vary if a child’s perspective were taken into account and the sample extended. Another limit of the study is that participants were limited in their answers only by the boundaries set up by the parenting concept. Thus, parents were free to provide a large array of activities and themes as answers referring to whatever they perceived as a parenting concept. Future research should investigate narrower concepts in parenting such as control, autonomy, or support, for example. Further research could also investigate the perceptions of parents and children comparatively and how time in forced isolation can be better used to develop parenting skills and child wellbeing. As the present research found that children mostly lacked social interactions in the quarantine period, and online interactions for educational purposes increased, further research may evaluate the incidence of other healthy or risky behaviors in children as well, such as play activities among siblings or internet use, only if opportunities arise and new quarantine periods are decreed. Further research could also report children’s voice and their emotions during crisis times such as this. At the same time, future research should investigate how such critical periods should benefit from social work and psychological systematic interventions in order to make the period easier to pass [47,48].

## Figures and Tables

**Table 1:** The categories and subcategories of answers to the first research question of the qualitative study.

Categories and Subcategories	%	%C ^1^
1. Free Time Activities Interactive games, e.g., family members played Rummy, puzzle games, crossword puzzles… Entertainment activities, i.e., watching movies, painting, music, drawing… Sports and recreational activities, i.e., physical effort, e.g., table tennis, walking, dancing, forest walks… Time for self, e.g., personal care, praying, meditating… Reading books, studying	47.62 ^2^	47.62
	15.93	
	2.38	
	6.12	
	15.87	
	7.32	
2. Chores Inside, i.e., cooking, home reconstruction and redecoration, daily chores, painting walls, ironing Outside, i.e., shopping, cutting and painting trees, planting trees, agricultural activities	19.59	67.21
	15.75	
	3.84	
3. Relationship Preoccupations Interpersonal knowing each other better, i.e., granting attention to each child individually, religious fellowship, monitoring children better, improving relationships Being affectionate to each other, i.e., expressing love to each other, more affectionate physical contact with children, e.g., holding hands…, having more patience Talking, i.e., discussing calmly with children, small talk, talking about solutions to old problems Care and protecting children, i.e., organizing family life better, putting children to sleep at noon, caring for children’s needs	13.53	80.74
	3.66	
	2.19	
	6.22	
	1.46	
4. Exclusive online schooling, i.e., doing homework for a longer time with children, figuring out how to do online schooling, helping children with homework as teachers indicated	6.04	86.78
5. Religious activities, i.e., reading the Bible, praying, singing together, fellowship, online church	4.57	91.35
6. Unspecified activities, e.g., family routine, activities together…	3.47	94.82
7. No differences at all, i.e., parents did not report any better activities or changes during the quarantine time as compared to the time before	2.93	97.75
8. Hygiene activities regarding COVID-19, e.g., house disinfection, washing hands frequently and correctly, shoes and clothing disinfection	1.46	99.21
9. Money management, i.e., being cautious with expenses, being thankful for the present resources, earning more money	0.79	100

Legend: ^1^ %C = cumulative percent; ^2^ all numbers in the table are rounded.

**Table 2:** The categories and subcategories of answers to the second research question of the qualitative study.

Categories and Subcategories	%	%C ^1^
1. Difficulties in Adapting to the New, Imposed Social Reality Social distancing (i.e., impossibility to meet the loved ones, to visit grandparents, parents or cousins, no meetings at all) Moving outside the home (i.e., restrictions when going shopping, going out in nature, no traveling or leaving the restricted area, interdiction to go out with children) Respecting norms referring to COVID-19 (i.e., wearing medical masks, filling out the mandatory declaration, disinfecting objects, strict hygiene rules)	37.85 ^2^	37.85
	19.04	
	17.09	
	1.72	
2. Difficulties in Emotional Regulation (i.e., psychological tiredness, emotional insecurity, stress, boredom of children, depressive or anxious thoughts, no patience)	12.7	50.55
3. Online Schooling (i.e., less teacher support, no internet or computers, no piano or canto lessons, no kindergarten, technological problems with online homework, no classes for some school subjects)	9.71	60.26
4. Motivating Children to Comply (i.e., difficulties in explaining to children what quarantine and isolation mean, doubts and lack of motivation from children to stay indoors, no motivation for any school activity, no motivation for schedules, routines, or discipline over the day)	7.23	67.49
5. No Difficulty (i.e., no difficulty at all, life was as before)	6.09	73.58
6. Difficulties in Time Management (i.e., difficulties in establishing a study schedule for children, sleep management, waking up in the morning)	5.14	78.72
7. Living in a Small Dwelling (i.e., crowded house, being fed up living inside, being trapped inside)	3.61	82.33
8. Money Management (i.e., tendency to buy online more, investing too much in games and toys, lack of money for dwelling improvements)	3.42	85.75
9. Relationships Management (i.e., no ideas for activities with children, sibling conflicts, impatient children)	3.04	88.79
10. Home Chores (i.e., more cooking, sharing chores efficiently, finishing chores)	2.28	91.07
11. Hard Time Doing Physical Effort (i.e., no sport, children are agitated due to the lack of physical effort, no playing outside)	2.28	93.35
12. Media Devices Management at Home (i.e., too much time in front of screens, too many movies and cartoons, internet dependency risk)	1.9	95.25
13. Working at Home (i.e., no time for children as we both work at home, no room for moving job duties at home, difficulties to concentrate on job)	1.33	96.58
14. No Religious Activities Participation (i.e., no weddings, no church-going, no religious events)	1.33	97.91
15. Health Issues Restricting access to medical services (i.e., no access to dentists, online consulting, therapy for sick children) Health problems other than COVID-19 (i.e., physical discomfort, older health problems that needed care, lack of pills for health problems)	1.14	99.05
	0.76	
	0.38	
16. Eating (i.e., eating between meals, too much eating, no schedule for meals, no time for breakfast as every family member woke up at a different hour)	0.95	100

Legend: ^1^ %C = cumulative percent; ^2^ all numbers in the table are rounded.

**Table 3:** The categories and subcategories of answers to the third research question of the qualitative study.

Categories and Subcategories	%	%C ^1^
1. Complaints about their need for socialization (i.e., children cannot meet friends or school colleagues, they cannot visit grandparents and cousins, desire to see people and walk on the streets full of people)	46.93 ^2^	46.93
2. Interdiction to Move Outside their Property (i.e., children cannot travel by car, cannot go shopping, in the neighborhood, in the park, to the Mall, to the cinema, to a restaurant, or to the countryside…)	22.52	69.45
3. Schooling Complaints (i.e., kindergarten is shut down, online homework is boring, no access to the internet and computers, worries concerning exams, missing school, no extracurricular activities, concerns about evaluation and marks)	14.47	83.92
4. Emotion Management (i.e., fear of getting infected, boredom, daydreaming about life before the pandemic, stress, worries when hearing about bad news)	4.68	88.60
5. Lack of Sports Activities (i.e., no basketball, football, cycling, swimming, or group sports…)	3.51	92.11
6. Life Disturbance in relation to COVID-19 (i.e., children keep asking when everything will be fine again, why they must wear masks and gloves, it is not fair to cancel all activities, no fun at all, fed up with isolation…)	2.78	94.89
7. No Complaints at All (i.e., children played outside in the yard and had no complaints, they understood the situation, enjoyed nature…)	2.19	97.08
8. Food Complaints (i.e., no favorite foods, no special ingredients, no sweets…)	1.32	98.40
9. Religious Complaints (i.e., no access to church, too much prayer, missed the church atmosphere)	1.02	99.42
10. Time Management (i.e., no established schedule, exaggerating with activities, too much phone talk)	0.58	100

Legend: ^1^ %C = cumulative percent; ^2^ all numbers in the table are rounded.

## Data Availability

Not applicable.
